# Epidural administration of neostigmine-loaded nanofibers provides extended analgesia in rats

**DOI:** 10.1186/s40199-014-0073-6

**Published:** 2014-11-18

**Authors:** Masoomeh Yosefifard, Majid Hassanpour-Ezatti

**Affiliations:** Department of Biology, Sciences School, Shahed University, Tehran, IRAN

**Keywords:** Neostigmine, Nanofibers, Analgesia, Epidural, Electrospinning

## Abstract

**Background:**

In this study, neostigmine-loaded electrospun nanofibers were prepared and then their efficacy and duration of analgesic action were studied after epidural administration in rats by repeated tail flick and formalin tests.

**Methods:**

The neostigmine poly vinyl alcohol (PVA) nanofibers were fabricated by electrospinning methods. The nanofibers (1 mg) were injected into the lumbar epidural space (L5-L6) of rats (n = 6). Cerebrospinal fluid samples of rats were collected 1, 5 and 24 hours after injection and then were sampled once weekly for 4 weeks. Free-neostigmine concentration was measured in the samples spectrophotometrically. Rat nociceptive responses were evaluated by repeated tail-flick and formalin tests for 5 weeks after the nanofibers (1 mg) injection. Locomotor activity of rats was measured in the open-field at the same period.

**Results:**

The cerebrospinal fluid concentration of free neostigmine reached 5 μg/ml five hours after injection and remained constant until the end of the experiments. The tail-flick latency of treated rats was significantly (p < 0.01) increased and remained constant up to 4 weeks. Pain scores of the rats in both phases of formalin test were significantly (p < 0.01) reduced during the same periods, Epidural injection of the nanofibers had no effect on locomotor activity of rats in an open-field.

**Conclusions:**

Our results indicate that the neostigmine nanofibers can provide sustained release of neostigmine for induction of prolonged analgesia after epidural administration. High tissue distribution and penetration of the nanofibers in dorsal horn can increase thermal and chemical analgesia duration without altering locomotor activity in rats for 4 weeks.

## Background

Recently, researchers have been employing new techniques to improve both the efficacy and duration of analgesic effect of some drugs. Epidural administration of neostigmine could reduce pain in patients with uncontrolled pain [1]. It has been reported that intrathecally administered neostigmine could also provide effective analgesia in both phases of formalin test in rat [2]. Although, in clinical studies intrathecal neostigmine infusion is used for induction of prolonged analgesia in chronic patients [3], the application of a catheter for drug infusion would increase the risk of infection in patients and also requires complicated surgery. An alternative procedure for increasing the duration of neostigmine action after epidural injection is its combination with other drugs [4]. However, this method might induce side effects such as nausea, vomiting, sedation and respiratory depression in patients. It is shown that elevation of endogenous acetylcholine level at spinal cord synapses mediate neostigmine analgesia following epidural injection [5]. Also, intrathecal co-administered of neostigmine with local anesthetic can increase its duration of action [6]. Liposomal neostigmine for epidural application is another approach being used for control release of neostigmine, but unfortunately this formulation has short duration of action [7]. In recent years, incorporation of drugs in electrospun nanofibers has been used for making sustained and controlled-release drugs; Tseng and coworkers could fabricate lidocaine biodegradable nanofiber and showed a sustain delivery of lidocaine into the epidural space in rats [8]. On the other hand, it has been shown that intratecal administration of a dose of an analgesic drug could produce different results in the tail-flick and formalin tests [9]. Therefore, scientists compared the epidural or intrathecal anesthetic efficacy of same doses of an analgesic compound with two kinds of nociceptive stimulus such as tail flick and formalin test. Thus, it is certain that the selected dose of the drug is effective for different kinds of pain.

The aims of present study were: (1) fabrication of neostigmine-loaded poly vinyl alcohol (PVA) nanofibers by electrospinning methods; (2) *in*-*vitro* evaluation of neostigmine release from the nanofibers; (3) assessment of free-neostigmine concentration in the cerebrospinal fluid of rats after the nanofiber injection for 4 weeks; and (4) evaluation of the efficacy and duration of analgesia in thermal and chemical pain model by consecutive tail-flick and formalin test during 5 weeks after the injection of nanofibers.

## Materials and methods

### Chemical

Neostigmine methylsulfate was obtained as a gift sample from the laboratory of Dr. Sayyed Omid Ranaei Syadat, Tehran, Iran. In our experiment, all chemicals were of analytical grade purchased from Sigma-Aldrich.

### Preparation of nanofibers using electrospinning

**Figure 1:**
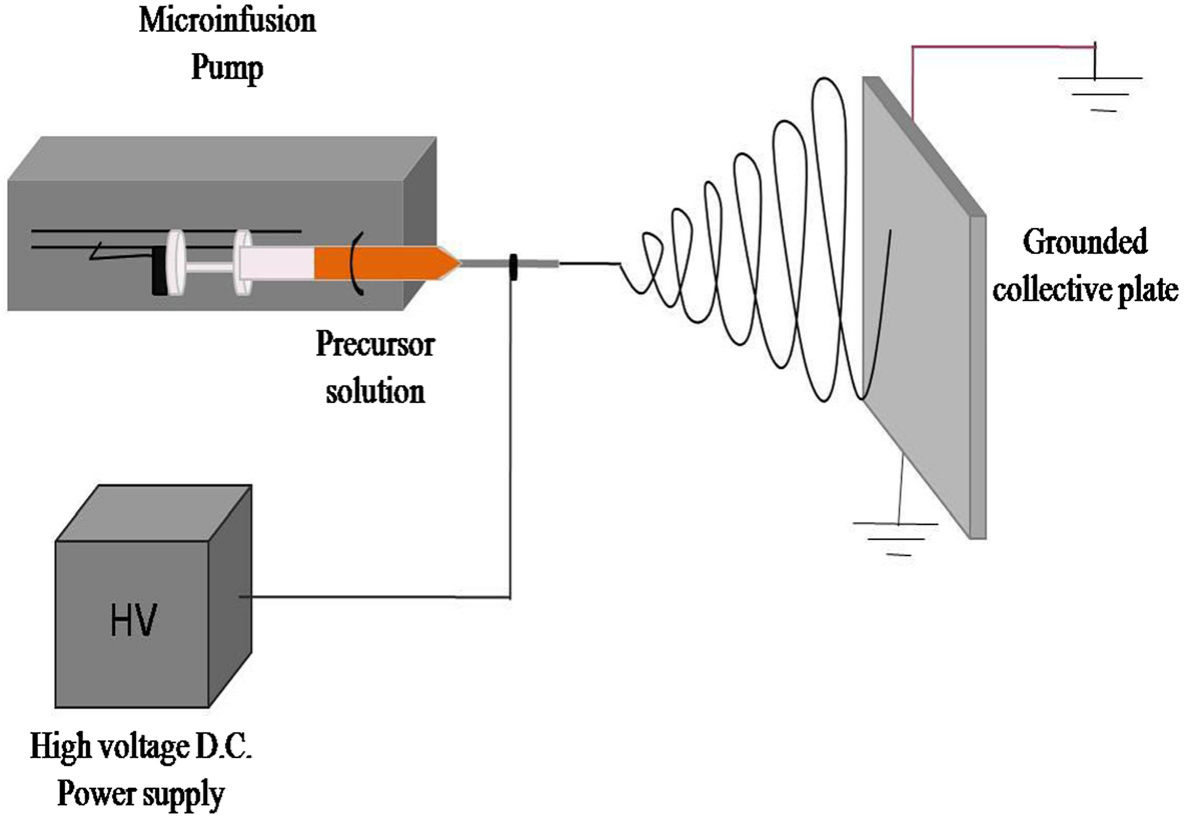
**Schematic representation of an electrospinning setup for production of neostigmine-PVA loaded nanofibers** [12].

The neostigmine-loaded poly vinyl alcohol nanofibers were prepared according to the procedure of Arecchi et al. [10]. Briefly, poly vinyl alcohol solution was prepared by dissolving 6 gram of poly vinyl alcohol powder in 100 ml of deionized water. The mixture was slowly heated to 95°C for 8 hours. To compensate for the loss of water due to evaporation during heating and stirring, deionized water was added to the solution to return it to the original volume. Thus, the final concentration of poly vinyl alcohol in solution was kept at 6 wt% [10]. Neostigmine 1.25% (w/w) was dissolved in double distilled deionized water and was added to the poly vinyl alcohol solution and stirred for 25 minutes before electrospinning. For preparing neostigmine-loaded electrospinning nanofibers, the 6% PVA solutions were mixed by volumetric ratios of 50:50 with neostigmine solution. Then, the mixture was pumped at a constant rate using a syringe pump toward a needle tip. The utilized electrical potential for electrospinning was 25 kV and the distance between the collector and the needle tip was 15 cm. The electrospinning was performed at room temperature and the resulting neostigmine-loaded nanofibers were collected on an aluminum foil. The schematic figure of electrospinning set up is shown in Figure 1. Then, the nanofibers were incubated at 150°C for 5 minutes, treated with ethanol for 1 hour and dried overnight at room temperature [11]. Final neostigmine-loaded nanofibers contained %1.25 (w/w) neostigmine/poly(vinyl alcohol). Finally, the structure of free and neostigmine-loaded nanofibers was studied using scanning electron microscopy.

### In vitro Acetylcholinesterase (AChE) inhibition assay by nanofibers containing different ratios of neostigmine/poly (vinyl alcohol)

The measurement of AChE inhibitory activity of neostigmine released from the nanofibers was carried out in a vial using spectrophotometric method proposed by Ellman et al. [13]. The percent of AChE inhibition was compared among the nanofibers that were made from different proportions of poly vinyl alcohol (5, 6, 7, 8%w/w) and contained different concentrations of neostigmine (0.000125 and 0.00125w/w). Acetylcholinesterase, AChE (E.C 3.1.1.7) was expressed with the baculovirus system [14]. A typical run consisted of 5 μL of AChE solution at final assay concentrations of 0.03 U/mL; 200 μL of 0.1 M phosphate buffer (pH 7.4); 5 μL of DTNB at a final concentration of 0.3 mM, prepared in 0.1 M phosphate buffer (pH 7.4) with 0.12 M of sodium bicarbonate. Then, the nanofibers containing neostigmine were added to each mixture reaction (1 mL) in vials, vortexed for 1 min, and then centrifuged rapidly at 16 000 g for 1 min. The intensity of the yellow solution in the resulting supernatants were determined spectrophotometrically at 412 nm every 5 min, three times consecutively. Percent of remaining activity of AChE was determined after incubation with the neostigmine-loaded nanofibers which were fabricated by different concentrations of poly vinyl alcohol and loaded with different doses of neostigmine.

### Encapsulation efficiency and in vitro release

To determine the encapsulation efficiency, 10 mg of the neostigmine-loaded nanofibers were stored in 1 ml of PBS (pH 7.4). The solution was incubated for 1 min at 37°C. At 5 min intervals, a sample was withdrawn and centrifuged at 16,000 g for 10 min. The precipitated samples were taken and resuspended in 10 ml fresh release medium to keep a complete sink condition and placed back to the shaker. The supernatant solution was retained for HPLC analysis. A mixture of acetonitrile and ammonium acetate (75:25 v/v) was added to the solution after the PBS had been removed. The resulting solution was analyzed using HPLC, in which a C-18 column was used and the mobile phase was delivered at a rate of 1 ml/min. One hundred microliters of sample was injected by an auto-sampler and the column effluent was detected at 248 nm.

### Morphologies of electrospun nanofibers

The surface morphology of the nanofibers was observed by scanning electron microscopy (S-4800, Hitachi, Japan). The free poly vinyl alcohol and neostigmine-loaded nanofibers were placed on a stage and sputter-coated with carbon.

### Animals

Adult male Sprague–Dawley rats (200–250 g) were purchased from Razi Institute of Iran. The rats were kept at 22°C, 12 hour night/day cycle, and received tap water and food *Ad libitum*. The present study followed the ethical guidelines for investigation of experimental pain in conscious animals as well as the Institutional Animal Ethical Committee of Shahed University, formed under Committee for Purpose of Control and Supervision of Experiments on Animals (CPCSEA, Reg. No. PRC-115), approved by the pharmacologic protocols [15].

### The experimental groups

The experimental groups consisted of six rats. Rats were divided into: (a) control group in which, the rats received epidural injection of 5 μl normal saline solution; (b) sham group in which, the rats were treated with epidural injection of 1 mg poly vinyl alcohol nanofibers and flushed by 5 μl saline solution; (c) neostigmine-loaded nanofiber group in which, the rats were treated with epidural injection of neostigmine-loaded nanofibers (1 mg) and flushed by 5 μl saline solution. Epidural injection was done at L5-L6 intervertebral space.

### Lumbar epidural injection of the neostigmine-loaded nanofibers

Rats were anesthetized briefly with ether and shaved at the lower back, then placed in the prone position with lower back elevated and flexed ventrally. A lumbar puncture was performed at L5–L6 intervertebral space, perpendicular to the skin, using a 30-gauge needle attached to a 50-μl Hamilton syringe. A catheter of a PE10 polyethylene tube, which was pre-filled with 1 mg neostigmine-loaded nanofibers isolated from 5 μl of saline using a small air bubble, was placed into the needle and advanced 4 cm from the tip of the needle up to the lumber enlargement, which was confirmed by a tail-twitch. The nanofibers were slowly injected and flushed with saline. Three minutes later, the catheter and the needle were respectively removed.

### Methylene blue injections

Pilot experiments were performed to evaluate the spread of injected solution in the spinal subarachnoid space. Using the same technique and injection volumes described above, the rats received spinal injections of 5 μl methylene blue 1 mg/ml solution. The area of spread of the methylene blue was examined upon animal necropsy 10 min after intrathecal injections. Besides, all intrathecal solutions contained 5% methylene blue and the included data were only from the animals in which intrathecal placement was confirmed postmortem.

### CSF sampling

CSF samples were collected based on procedures described by Haddadi et al. Rats anesthetized by i.p. injection of a mixture of ketamine (80 mg/kg) and xylazine (10 mg/kg) [16]. Then, each rat was placed in a stereotaxic apparatus and its neck was flexed so that a 29-gauge needle could be lowered between the base of the skull and the first cervical vertebra into the cisterna magna. Needle placement was verified by drawing a small amount of CSF into the needle and observing the clear CSF in the polyethylene tube connecting the needle to a 100 μl syringe (Hamilton). CSF (50 μl) was collected in polypropylene test tubes that were put immediately on dry ice and then stored at −80°C until the determination of neostigmine concentration. Samples of CSF of rats were taken 1, 5 and 24 h after the injection of neostigmine nanofibers. The sampling procedure continued once a week for 4 weeks.

### Measurement of neostigmine concentration in CSF of rats

Neostigmine concentration in CSF sample of rats (n = 6) was estimated by ultraviolet visible spectrophotometer at 261 nm (Shimadzu UV-1700, Japan) [17]. Aqueous standard solutions of neostigmine methylsulfate were prepared in phosphate buffer (pH 7.4) and their absorbance was measured by applying the same procedures. The method was validated with respect to precision and linearity. To determine the precision of the method, neostigmine concentration in CSF samples was analysed six times a day (intra-day precision) and during six continuous days (inter-day precision). The linearity of measurement was evaluated by analyzing different concentrations of the standard solution of neostigmine. Beer-Lambert’s concentration range was found to be 0.01-0.001 μg/ml [17].

### Behavioral tests

In the present study, two methods were simultaneously used for evaluating anesthetic effect of a single dose of neostigmine-loaded nanofibers against thermal and chemical types of nociceptive stimuli after epidural injection. Some scientists have claimed that the analgesic effect due to the activation of cholinergic mechanisms also depends on the experimental pain model that is utilized for its evaluation [18].

### Tail-flick test

The repeated tail-flick method was used for measuring the analgesic responses of rats after epidural treatment with the neostigmine-loaded nanofibers to a high intensity thermal nociceptive stimulus. Some researchers have also suggested that changes in tail-flick latency may be interpreted in terms of central sensitization and that the repeated tail-flick latency might be considered as a useful marker of chronic nociception [19]. Thus, the tail-flick latencies were repeatedly measured in rats before and on 1^st^, 15^th^ and 21^st^ days after epidural injection based on the methods proposed by Kríz et al. [19]. The average time interval between the onset of light stimuli and the tail-flick response was measured and defined as tail-flick latency. Since the test should be conducted in triplicates, the tail was marked in three places: proximal, middle, distal. The intensity of radiant heat was adjusted to establish the baseline latencies for 3–5 seconds. The heat stimulus was discontinued after 20 seconds to avoid tissue damages. (Cut off point = 20 s). Each rat was tested 3 times with a 3-second interval. Data were expressed as mean ± SEM (n = 6).

### Formalin test

The formalin test is usually used for evaluation of anti-nociceptive drugs which are administrated intrathecally against the high intensity of chemical pain stimulus. In order to avoid interaction of both techniques’ effects on the same animals, it is better to evaluate the formalin test at least 7 days after the tail-flick test, because the tail-flick test has no impact on the formalin test results after this period [20]. Therfore, in this study the formalin test was performed 7 days after the tail flick test. In practice, formalin (2.5%, 50 μl) was injected subcutaneously into the intraplantar surface of different feet of rats after treatment with nanofibers. Then, the rats were gently placed in plexiglass chambers. The pain behavior within the first 15 min of intraplantar formalin injection was recorded as the early phase scores, while the pain behavior between 20 and 60 min of the formalin injection was recorded as the late phase. The behavioral rating scale was as follows: 0 = the injected paw is not favored; 1 = the injected paw rests lightly on the floor and little or no weight is placed on it; 2 = the injected paw is elevated and is not in contact with any surface; 3 = the injected paw is licked, bitten and shaken. Pain scores were calculated by using this formula:

$$ \mathrm{Pain}\ \mathrm{score}=0\mathrm{T}0+1\mathrm{T}1+2\mathrm{T}2+3\mathrm{T}3/\mathrm{Time}\ \mathrm{block}\left(\mathrm{s}\right) $$

where T0-T3 is the number of seconds spent in each of the behavioral categories. All tests were conducted between 9:00 _AM_ and 5:00 _PM_. Pain scores were expressed as mean ± SEM. A probability of p < 0.01 was considered significant.

### Measurement of locomotor activity in open field

Locomotor activity was measured using an open field test. The rats were individually placed in one corner of the open field (100 × 100 × 48 cm). Movement of each rat in the field during 15 min of testing session was recorded. After 15 min, the rat was removed to the home cage, and the open field area was cleaned. The total distance and the average velocity of each rat in the field were recorded.

### Statistical analyses

The results were expressed as mean ± S.E.M., and statistical significance was evaluated by a two-way repeated measure analysis of variance (ANOVA) followed by Bonferroni tests. The statistical significance criterion (P-value) was 0.05. All data calculations and statistical analyses were done by using Prism version 5 (GraphPad Software Inc., San Diego, CA).

## Results

### Morphology of electrospun nanofibers

**Figure 2:**
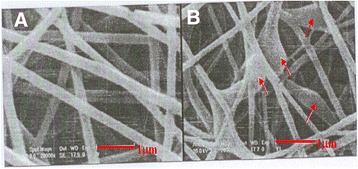
**The Scanning electron microscope photomicrograph of Polyvinyl Alcohol nanofibers before (A) and after neostigmine loading (B).** In this figure the arrows show the loading position of neostigmine in the core of nanofibers. Magnification: 20,000 × .

According to the SEM micrograph shown in Figure 2, electrospun free poly vinyl alcohol nanofibers (A) and the neostigmine-loaded nanofibers (B) were circular in cross-section with an average diameter ranging from 500 nm up to 1,000 nm. The drug encapsulation in vesicular-like reservoirs along the nanofibers was confirmed by the scanning electron microscopy.

### *In vitro* AChE inhibition

**Figure 3:**
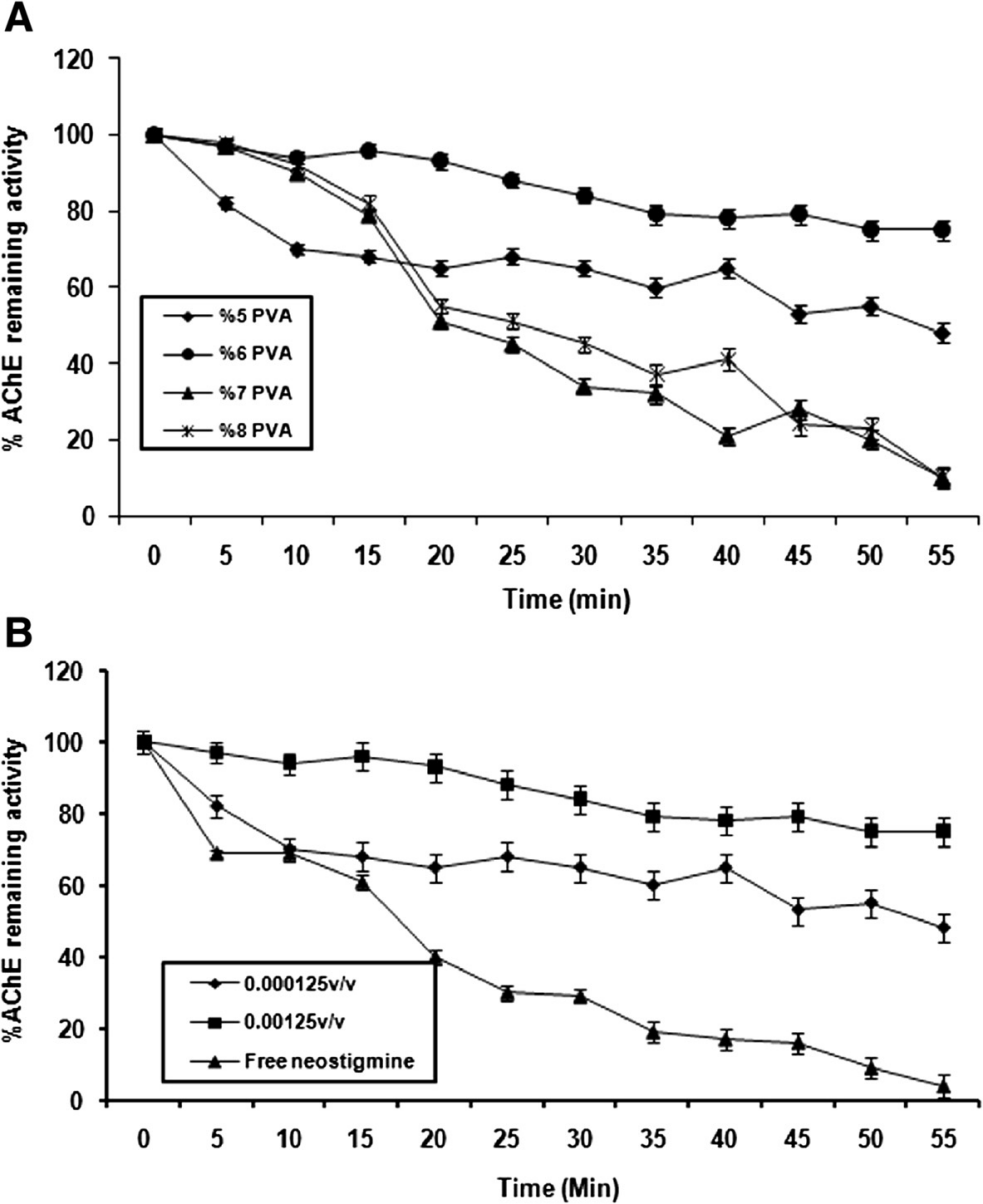
**Percentage activity remaining during inhibition of acetylcholinesterase after addition of nanofibers.** **(A)** The measurement of acetylcholinesterase activity following addition of the nanofibers were fabricated with different proportions of poly vinyl alcohol and neostigmine (0.00125 v/v); **(B)** the acetylcholinesterase activity after addition of the neostigmine nanofibers that fabricated by %6 PVA and two different concentrations of neostigmine (pH = 7/4, 22°C).

The percent of AChE inhibition changed with proportions of PVC (Figure 3A) and the concentration of neostigmine (Figure 3B) that were used in fabrication of the loaded-nanofibers. All neostigmine-loaded nanofibers contained some levels of inhibitory activity against AChE. However, the nanofibers containing 6% poly vinyl alcohol and 0.00125w/w neostigmine showed more effective inhibitory effect on AChE activity compared to others in vitro.

### Verification of epidural injection

**Figure 4:**
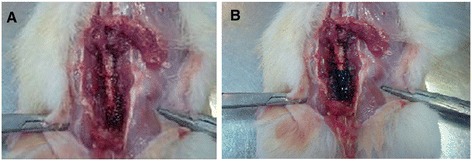
**Verification of injection site and evaluation the distribution of methylene blue injected into the epidural space.** **(A)** The exposed lumbar spinal cord region of control rats and **(B)** drug extension evaluation by epidural injection of 50 μL 1% methylene blue.

The spread of methylene blue dye into the epidural space indicated that the dye was only distributed in lumbar segments of rat spinal cord (Figure 4).

### Neostigmine concentration in CSF of rats

**Figure 5:**
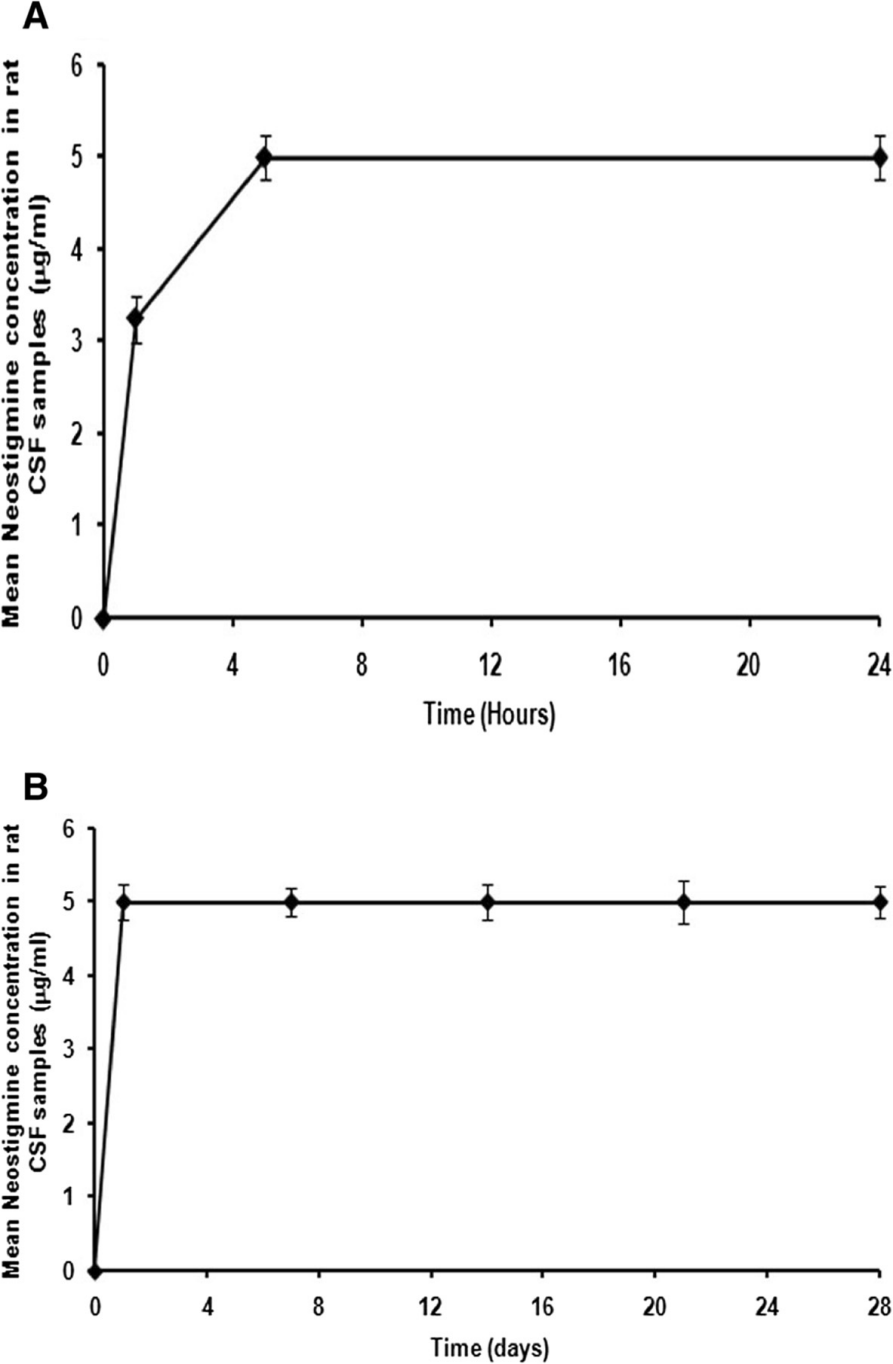
**Measurement of neostigmine concentration in CSF in rats.** CSF concentrations of neostigmine were measured, **(A)** up to 24 hours and **(B)** for 4 weeks after epidural injection of neostigmine loaded nanofibers. Values presented are means ± SEM.

The time course of neostigmine concentrations in CSF is shown in Figure 5. The results of this study showed that neostigmine concentrations in CSF were significantly increased from baseline to a maximum concentration (5 ± 0.1 μg/ml) during 5 hours after epidural administration (Figure 5-A). The concentration of free neostigmine in CSF of rats remained constant at 5 ± 0.1 μg/ml during 4 weeks after injection of the nanofibers (Figure 5-B).

### Formalin test

**Figure 6:**
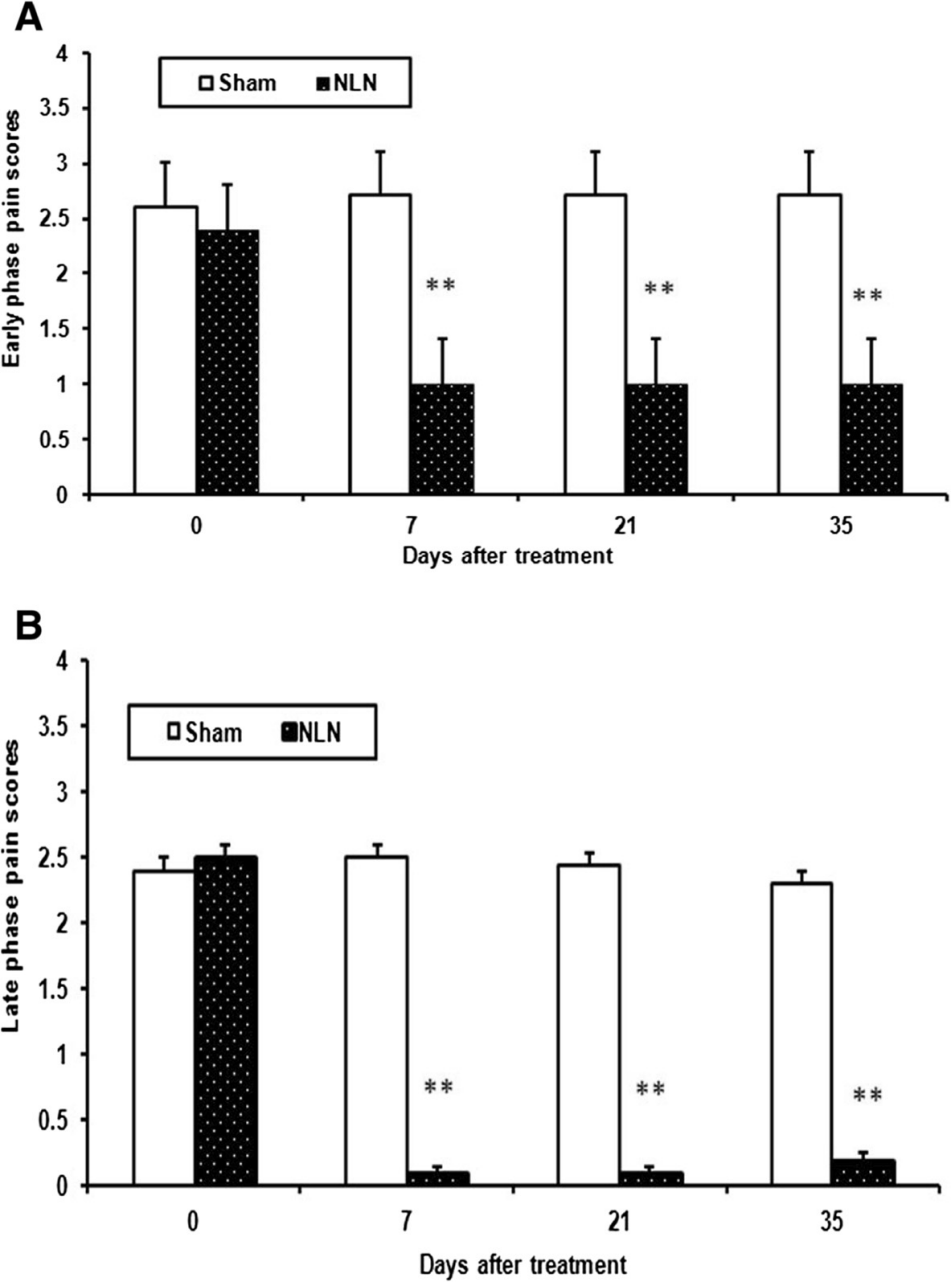
**The effect of epidural injection of neostigmine loaded nanofibers (NLN) on the cumulative nociceptive scores of rats in the early phase (A) the late phase (B) of formalin test.** Sham group treated with free PVA nanofibers (Mean ± SEM, n = 6, **p < 0.01, Repeated-two way ANOVA followed by Bonferroni test).

The epidural administration of neostigmine-loaded nanofibers decreased the pain score significantly (p < 0.001) in early (Figure 6A) and late (Figure 6B) phases of the formalin test in rats for five weeks after injection of the neostigmine nanofibers.

### Tail-flick test

**Figure 7:**
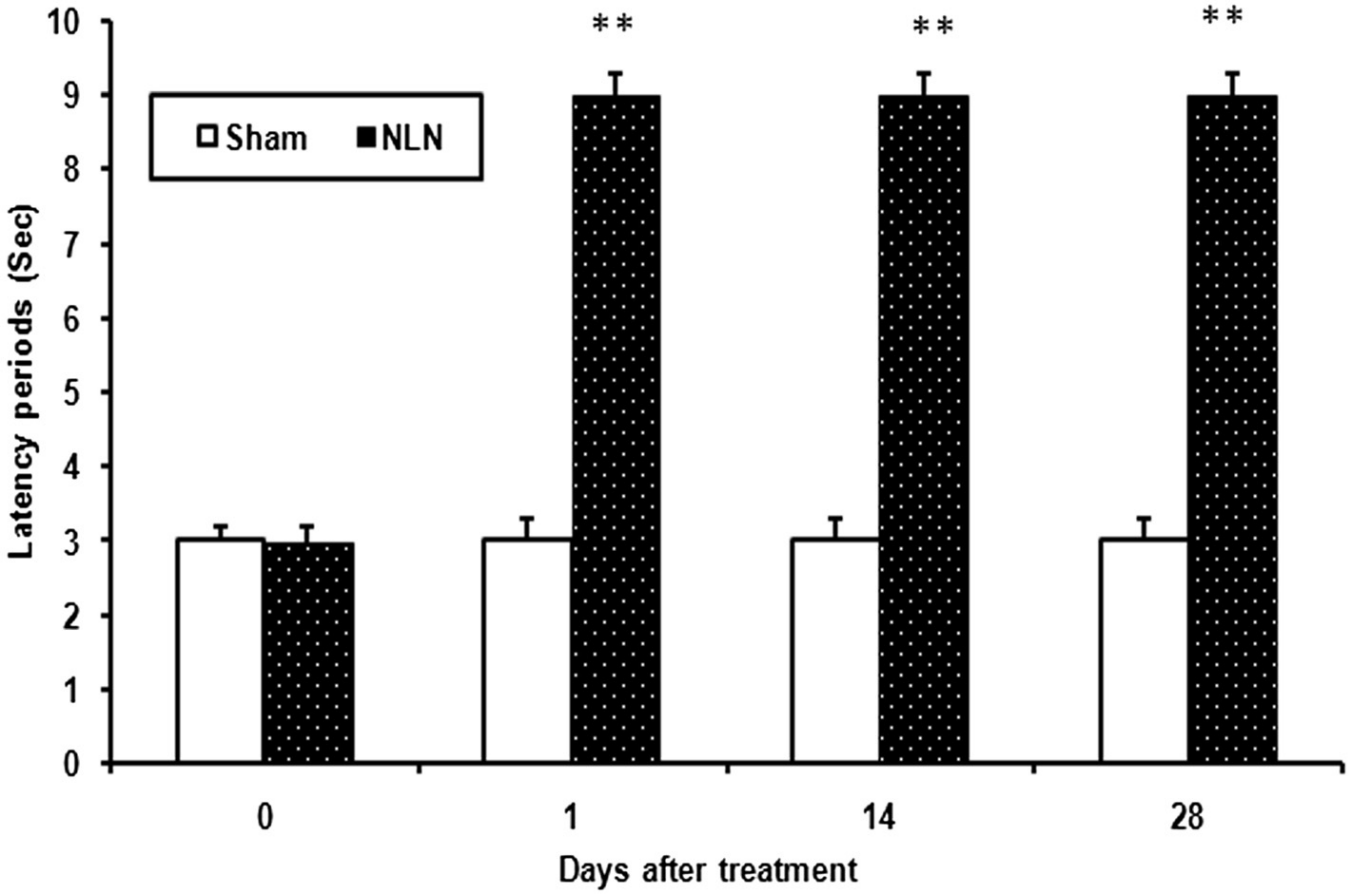
Effect of epidural neostigmine loaded nanofibers (NLN) administration on tail-flick latencies of rats (Mean ± SEM, n = 6, **p < 0.01, repeated two way ANOVA followed by Bonferroni test), Sham group treated with epidural free PVA nanofibers.

The duration of analgesia after epidural injection of neostigmine-loaded nanofibers is shown in Figure 7. The tail-flick latency of rats was significantly (p < 0.01) increased and remained stable for four weeks after injection of the neostigmine nanofibers.

### Locomotor activity in open-field

**Figure 8:**
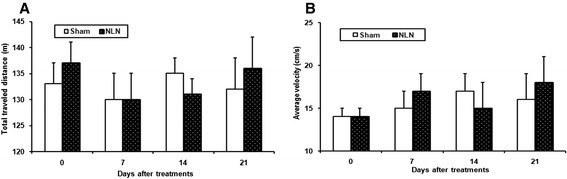
**Measurement of rats total traveled distance (A) and average velocity (B) for 3 weeks after epidural injection of neostigmine loaded nanofibers (NLN).** Sham group treated with free PVA nanofibers (Mean ± SEM, n = 6, **p < 0.01, Repeated-two way ANOVA followed by Bonferroni test).

The mean traveling distance (Figure 8A) and the velocity (Figure 8B) of rats in the open-field in 7, 14 and 21 days post-injection were not affected by epidural administrations of the nanofibers in comparison with the sham group.

## Discussion

The results of the present investigation revealed that neostigmine was successfully loaded into the poly vinyl alcohol nanofibers using the electrospinning technique. The SEM images confirmed the incorporation of neostigmine in the nanofibers. As seen in Figure 2, no drug crystals were detected by electron microscopy on the surface or outside of the loaded nanofibers. The electrospinning technique has been already used effectively to produce other drug-loaded nanofibers. The poly vinyl alcohol nanofibers have proven their performance in controlled release of antinociceptive drugs [21]. PVA nanofibers prepared by electrospinning technique also provide a suitable matrix for sustain release of drugs based on available evidence [22]. Therefore, it seems that the electrospinning method and poly vinyl alcohol nanofibers are appropriate choices for encapsulation of neostigmine.

The in vitro study of AChE inhibition after injection of the nanofibers indicated that the neostigmine released from the nanofibers can inhibit AChE effectively and the loading of neostigmine within the nanofibers did not compromise the inherent of AChE inhibitory activity. The results of enzyme activity assay also indicated that the change in the ratio of PVA- neostigmine can influence the amount of neostigmine release and percent of AChE inhibition. Measurement of neostigmine concentrations in cerebrospinal fluid (CSF) has suggested that the profile of the neostigmine release from the loaded nanofibers follows a biphasic pattern characterized by an initial fast release during 5 hours and following sustained release phase. A similar biphasic behavior has also been reported for ethyl cellulose nanofibers fabricated by using electrospinning process [23]. This form of drug release is an ideal situation for the rapid relief of symptoms which optimizes the therapy and avoids repeated administration for the patients’ convenience. Furthermore, the researchers treated the nanofibers with ethanol in order to reduce the drug release rate. It is reported that the burst release of drug from nanofibers was eliminated after treatment with alcohol. In practice, the use of such drug-loaded nanofibers for spinal cord drug delivery can increase the duration of drug efficacy. For example, Schmidt and co-workers enhanced epidural liposome-encapsulated hydromorphone’s duration of analgesia action from 2 to 72 hours in rats [24]. Based on the above reports, a combination of all these mechanisms can provide explanation for stable concentration of neostigmine in in vitro situation.

The results of the present study indicated that the thermal pain threshold was increased in rats after single epidural injection of the neostigmine-loaded nanofibers and persisted for as long as 28 days. Consecutive formalin test in rats also showed that the pain scores decreased in both phases and remained stable up to 35 days after injection. The early phase response of the formalin test was considered to be the result of direct effects of formalin on nociceptive fibers. It may be modulated by cholinergic spinal inhibitory interneurons [25]. The late phase was caused by tissue damage as well as inflammation and reflecting a state of central sensitization. Park and co-workers demonstrated that intratecal administration of atropine, a muscarinic antagonist, could decrease rat hindpaw persistent pain after formalin injection into the hindpaw [26]. Moreover, the intrathecal injection of neostigmine could reduce the pain in both phases of the formalin test.

The high sensitivity of tail flick technique for measurement of pain threshold after manipulation of spinal cholinergic system has been reported [27]. In addition, consecutive evaluation of tail-flick latency was considered as a marker of chronic nociception. Comparing the anti-nociceptive effect of the neostigmine loaded nanofibers in these two pain models showed that the effective analgesic dose of neostigmine was chosen for pain relief in our experiments.

In support of the present findings it can be said that after being release from nanofibers, the free neostigmine increased the level of endogenous acetylcholine at dorsal horn of spinal cord. Following mechanisms have been proposed to explain the anti-nociception action of acetylcholine at the dorsal horn level. It is shown that acetylcholinesterase inhibition increased acetylcholine level at spinal cord and then it caused (i) the activation of presynaptic nicotinic acetylcholine receptors and decrease in glutamate release from C-fiber terminals in the dorsal horn [28]; (ii) the inhibition of presynaptic release of glutamate from primary afferent axons by stimulation of GABAergic interneurons via activation of muscarinic acetylcholine receptors [29]; (iii) the stimulation of the nicotinic acetylcholine receptors on GABA-ergic inhibitory interneurons that triggered the firing of spinothalamic pain transmission neurons [30]; and (iv) the reduction of neuroinflammation by blocking microglial cell activity via stimulation of the nicotinic receptors [31]. Furthermore, neostigmine could stimulate GABA release from dorsal horn neurons [32] and its stimulatory effect on GABA release continued even after drug clearing from the CSF [33]. All of these mechanisms can play a significant role in effective analgesia induced by epidural application of neostigmine nanofibers.

In addition, loading of drugs in nanostructures increased its tissue penetration power [34], which probably makes them activate more inhibitory interneurons in deeper layers of dorsal horn of spinal cord. This property could provide an additional advantage for neostigmine-loaded nanofibers.

Comparison of the present findings with previous results [3,35] showed that the duration of analgesia after administration of the neostigmine-loaded nanofiber was longer than all previous neostigmine-containing mixture in animal or human studies. A relation was shown between concentrations of a drug in cerebrospinal fluid and its analgesic effect [36]. It can be concluded that the loaded amount of neostigmine in the nanofibers was sufficient to produce and maintain a relatively constant concentration of free neostigmine in CSF for induction of long-term analgesia. Also, the low rate of drug biotransformation in CSF could be another possible factor to increase the duration of neostigmine effect after epidural injection [37].

In spite of the free neostigmine side effects on locomotor activity after epidural administration in human [38], injection of the neostigmine-loaded nanofibers had no adverse effects on rats. Furthermore, the epidural administration of neostigmine-loaded nanofibers did not significantly alter locomotor activity in rats. Probably, the lack of side effects is due to the restricted release of neostigmine from nanofibers in dorsal horn of spinal cord. In support of this idea, it was shown that no adverse effects were noted after the epidural administration of neostigmine when the drug spread was restricted into lower part of the spinal cord [39].

## Conclusion

In this study, electrospun polyvinyl alcohol nanofibers were used as a controlled release matrix for the incorporation of neostigmine. The findings suggested that the nanofibers made from poly vinyl alcohol can easily be loaded with neostigmine. The SEM image confirmed loading of drug in nanofibers. The lumbar epidural administration of the nanofibers can reduce acute and chronic thermal and chemical pain in rats for 5 weeks. Also, its application had no significant effect on locomotor activity of rats.

## Abbreviations

AChE: Acetylcholinesterase
PBS: Phosphate Buffer Saline
CSF: Cerebrospinal Fluid
NLN: Neostigmine Loaded Nanofibers
